# Is Ireland ready for tobacco endgame? A national survey of knowledge and attitudes to tobacco endgame

**DOI:** 10.1093/eurpub/ckac129.034

**Published:** 2022-10-25

**Authors:** E Cosgrave, M Blake, E Murphy, A Sheridan, F Doyle, P Kavanagh

**Affiliations:** 1 Department of Public Health HSE South East, Health Service Executive, Kilkenny, Ireland; 2 HSE Tobacco Free Ireland Programme, Health Service Executive, Dublin, Ireland; 3 Department of Health Psychology, Royal College of Surgeons in Ireland, Dublin, Ireland

## Abstract

**Background:**

In 2013 ‘Tobacco-Free Ireland’ (TFI) shifted Irish national policy from tobacco control to “tobacco endgame”: policies, plans and interventions seeking to end the tobacco epidemic completely. Recent trends suggest the current 2025 TFI goal will not be achieved. This cross-sectional study is a timely assessment of public knowledge and attitude to re-focus Irish “tobacco endgame” planning.

**Methods:**

A literature-informed, pre-tested survey instrument was telephone-administered to a representative sample of 1000 members of the Irish public aged ≥15 years recruited via random digit dialling in February 2022. Prevalence of “tobacco endgame” views was measured; logistic regression determined factors associated with key responses.

**Results:**

Response rate was 30% (n = 1,000, post-hoc weighting applied). While TFI goal awareness was low (34%), support was high (75%), albeit most (61%) recognised postponement beyond 2025 was required for achievability. There was majority support for 18/22 endgame measures assessed. Product-focused tactics were popular, while views on targeting users were mixed: e.g. 86% supported a reduction in tobacco-product nicotine content; 40% supported introduction of a tobacco-user license. Phasing-out tobacco sales was highly-supported (83%); however, this was contingent on special supports for those currently addicted. Support for the TFI goal was higher among non-tobacco users (aOR 2.66, 95%CI 1.89-3.76), females (aOR 1.57, 95%CI 1.17-2.11) and those of higher social class (aOR 1.72, 95%CI 1.25-2.35).

**Conclusions:**

While achievement by 2025 is increasingly unrealistic, findings strongly affirm Irish public opinion is ready for “tobacco endgame”. Recognition of the needs of currently addicted tobacco users and focusing on subgroups with lower support levels should be integral to equitable “tobacco endgame” planning and communication. This study should mobilise renewed Irish political commitment to bold actions aimed at ending smoking-related harm.

**Key messages:**

• There is strong support for tobacco endgame measures among the Irish population, which is a supportive factor for bold political leadership to make these radical ideas a reality.

• Public preference for product and non-user-focused measures aligns with tobacco endgame discourse and should aid policy reframing to tackle structures and dynamics sustaining the tobacco epidemic.

